# A Theoretical “Cure” for Cancer

**Published:** 1906-09

**Authors:** 


### EDITORIAL DEPARTMENT.

### A Theoretical "Cure" for Cancer.

**A Boom for Trypsin.**

One of the most remarkable papers I have seen lately is one in *McClure's* for August, by Dr. C. W. Saleely, of London, entitled: "Cancer; Can It Be Cured?" It is remarkable in that it was published in a popular magazine instead of in the medical press; remarkable for the absence of facts,—inferences, unwarranted, are taken for facts; and remarkable for the facility with which the writer jumps to the conclusion that trypsin will cure cancer, because two mice in which the writer alleged that he had induced cancer by inoculation, seemed to get well under the trypsin treatment. The most remarkable sentence in the paper, however, it seems to me,—and it accounts for the doctor's robust credulity,—is this: "If the cases I have seen, be not * * * due to divine interference with natural law, one has no choice but to speak."

"Oceans into tempests wrought, to waft a feather or drown a fly," says Young. The doctor believes that divine interference with natural law—to save an inoculated mouse, would be not so surprising. The alternative being that he, Dr. S., had cured the mouse. He therefore proceeds to "speak," and he speaks out loud and at length through *McClure's, to the people.*

It seems to me that he sets up a man and knocks him down to his own satisfaction.

The paper is based upon Dr. Beard's well-known theory of cancer, which has been so much discussed in the medical press lately, that I will not enter fully upon it here.

The doctor constitutes himself a kind of Boswell to Dr. Beard's Johnson, and proceeds to exploit Dr. Beard, his theory, himself, and *Trypsin.* At best, it is not even a rational guess, but it will undoubtedly have the effect of booming trypsin, creating a tremendous demand for and sale of that article (perhaps this was the underlying motive of the paper), and self-dosing by those who have, or think they have, cancer. Has *McClure* allowed itself to be worked? or—was it a paid-for advertisement in the interest of some English manufacturer of "animal extracts"? Why should Dr. S. go to the people with this fine-spun theory? They can not understand it; but they can understand that he clearly means to say that trypsin will cure cancer, and that will sell it. Beard himself has written more clearly and sensible about his experiments and observations, in the medical press (he said nothing about divine suspension of natural law, however), and so has Shaw McKenzie (*British Medical Journal*), and Gaylord (*Medical Record,* New York), and Clowes (*Surgery of Gynecology and Obstetrics*), and Ehrlich, of Frankfurt (*Zeitft. f. gerzliche Fortbildung.*)

The latest I have seen on this subject is the following from *The New York Medical Record*,—showing the bursting of the bubble. But this fact will not stop the sale of trypsin and the self-dosing of the unfortunates:

"FAILURE OF THE TRYPSIN TREATMENT OF CANCER.

"A special despatch to *The New York Times* states that an inquiry made regarding the use of trypsin as a cure for cancer in the London hospitals has elicited unfavorable reports. The London Cancer Hospital has discontinued the use of the remedy, the surgeons having failed to obtain any beneficial results from it. In some hospitals the experiments are still proceeding, but apparently without expectation that they will result otherwise than have the tests at the Cancer Hospital."

